# Superlubric Graphullerene

**DOI:** 10.1021/acs.nanolett.4c02794

**Published:** 2024-08-19

**Authors:** Penghua Ying, Oded Hod, Michael Urbakh

**Affiliations:** Department of Physical Chemistry, School of Chemistry, The Raymond and Beverly Sackler Faculty of Exact Sciences, and The Sackler Center for Computational Molecular and Materials Science, Tel Aviv University, Tel Aviv 6997801, Israel

**Keywords:** graphullerene, structural superlubricity, multicontact, density functional theory calculations, registry index, interlayer potentials, sliding energy corrugation

## Abstract

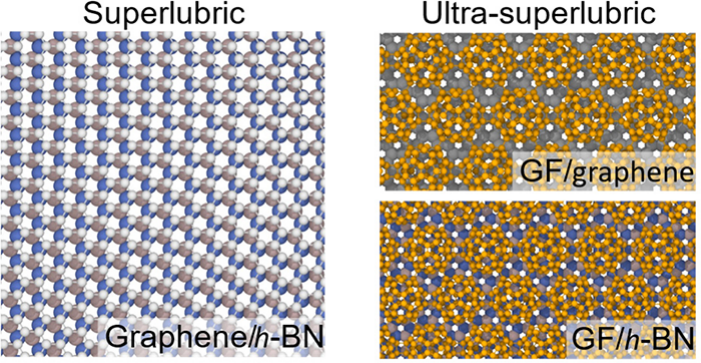

Graphullerene (GF), an extended quasi-two-dimensional network of C_60_ molecules, is proposed as a multicontact platform for constructing superlubric interfaces with layered materials. Such interfaces are predicted to present very small and comparable sliding energy corrugation regardless of the identity of the underlying flat layered material surface. It is shown that, beyond the geometrical effect, covalent interlinking between the C_60_ molecules results in reduction of the sliding energy barrier. For extended GF supercells, negligible sliding energy barriers are found along all sliding directions considered, even when compared to the case of the robust superlubric graphene/h-BN heterojunction. This suggests that multicontact architectures can be used to design ultrasuperlubric interfaces, where superlubricity may persist under extreme sliding conditions.

Friction is a ubiquitous phenomenon arising when two surfaces slide against each other, leading to energy loss and material wear. Traditionally, liquid lubricants are introduced into the interface to reduce friction. These, however, require heavy maintanance and fail under extreme environmental conditions. An alternative appoach that was demonstrated at the nano- and microscales involves layered material contacts. When stacked in an incommensurate configuration (where the periodicities of the contacting surfaces in each direction have an irrational ratio), the interfacial friction nearly vanishes (with friction coefficients below 10^–3^) due to effective cancellation of lateral forces, an effect known as structural superlubricity.^1−6^ In heterogeneous layered junctions, superlubricity was predicted^7^ and shown experimentally^8^ to be robust against interfacial reorientation due to the intrinsic lattice mismatch between the contacting surfaces. Nonetheless, the scaling up of superlubricity poses a number of challenges that have to be addresed, including elasticity effects, and the inveitable appearance of lattice defects^9^ and grain boundaries.^10^

A remedy for these can be obtained by a multicontact strategy that breaks down a macroscale interface into a large set of nano- or microscale incommensurate superlubric contacts.^4^ The recently synthesized graphullerene (GF)^11^ carbon allotrope, also known as quasi-hexagonal phase fullerene,^12^ offers a promising route for the fabriction of such multicontact junctions (see Figure 1b). Notably, GF exhibits high mechanical strength^13,14^ and thermal conductivity,^15^ which are important properties for interfacial lubrication. The unit cell of this extended sheet comprises of two C_60_ molecules, interlinked by one and two single C−C bonds (see Figure 1a). When interfaced with a two-dimensional material surface, this forms a set of well-defined atomic scale contacts separated by elevated regions. If most of the contacts form an incommensurate interface, scaling of friction with surface area is expected to be sublinear, and superlubricity is expected to prevail.

**Figure 1:**
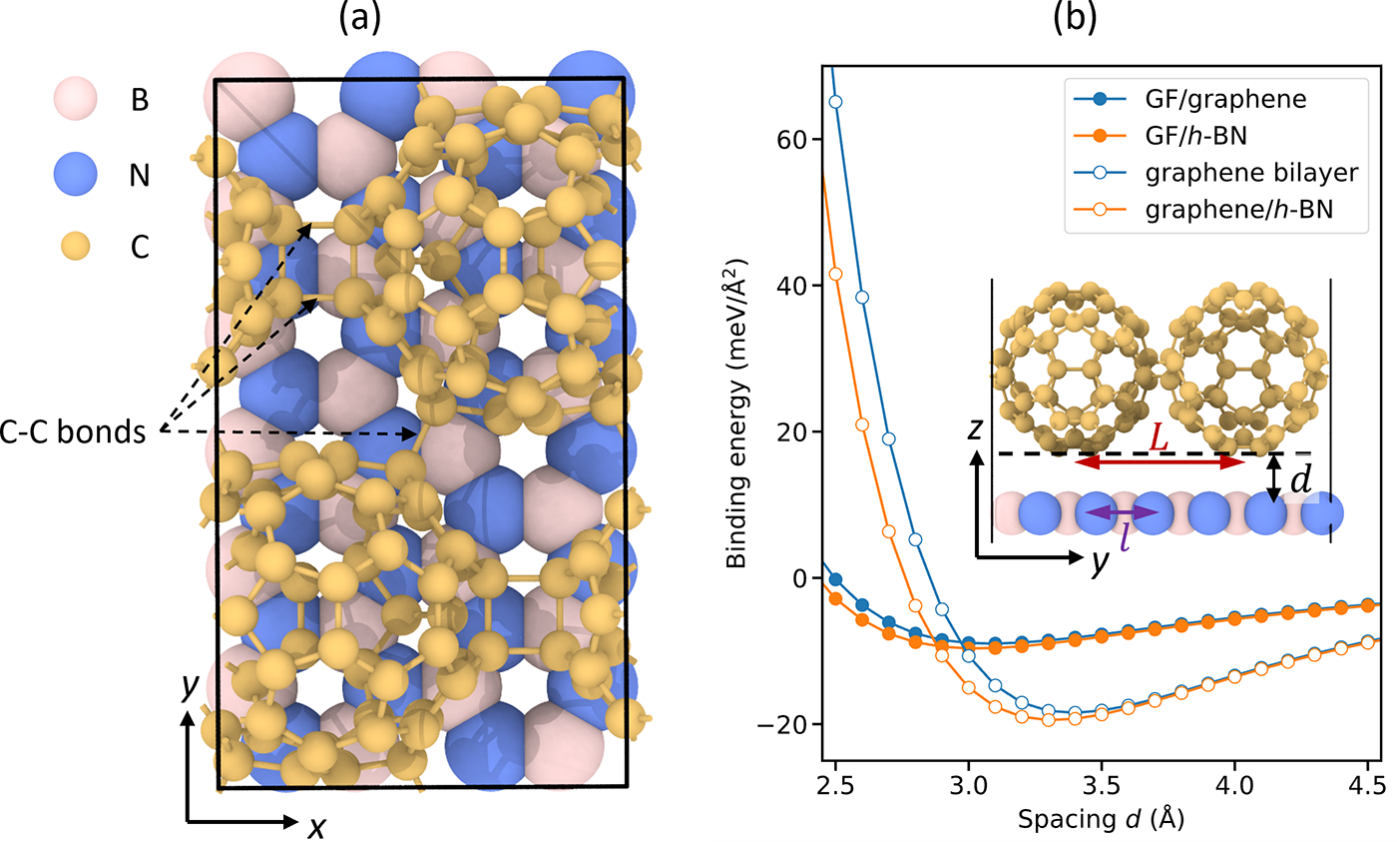
Heterojunction model system. (a) Top view of the rectangular GF (1 × 1)/*h*-BN (2 × 6) supercell containing 120 carbon (yellow spheres), 24 nitrogen (blue spheres), and 24 boron (pink spheres) atoms. (b) Binding energy curves of GF/graphene (filled blue circles), GF/*h*-BN (filled orange circles), graphene (empty blue circles), and graphene/*h*-BN (empty orange circles) bilayers. The inset shows the definition of the interlayer distance, *d*, as the vertical distance between the lowest GF atom and the plane of the flat substrate. Also shown is the distance between adjacent contact points, *L*, which is larger than the lattice vector of the underlying flat layer, *l* (*L* > *l*), thus forming a multicontact interface. The OVITO package was used for visualizing atomic structures.^16^

To validate this hypothesis, we investigate the sliding energy landscape of GF/graphene and GF/*h*-BN interfaces via first-principles calculations. To that end, we first performed separate structural relaxations of the GF and the underlying graphene or *h*-BN substrates (see optimized lattice constants in Table 1). A rectangular bilayer supercell of the average lattice constants of the two layers was then constructed, where the two parallel C−C bond bridges are aligned along the armchair axis of the substrate. This yields an intrinsic strain of ∼3.5% and ∼2.5% at the contacting surfaces of the GF/graphene and GF/*h*-BN heterojunctions, respectively. Structural relaxation of the atomic positions within the strained supercell was then performed. A top view of the resulting GF/*h*-BN heterostructure is presented in Figure 1a.

**Table 1:** Lattice Constants (in angstroms) of the Considered Bilayer Interfaces along the Armchair (*x*) and Zigzag (*y*) Directions of the Flat Surface^a^

2D crystals	armchair (*x*)	zigzag (*y*)
graphene	4.26	2.46
*h*-BN	4.34	2.50
GF	9.12	15.80
graphene bilayer	4.26	2.46
strained GF/graphene	8.82	15.28
strained GF/*h*-BN	8.90	15.41

a Rectangular supercells are used.

First-principles calculations were performed using density function theory (DFT) with the PBE exchange-correlation energy functional approximation^17^ and the Tkatchenko-Scheffler description of long-range dispersion interactions^18^ with many-body corrections,^19,20^ as implemented in the VASP package.^21^ Single-point calculations were performed using an energy cutoff of 650 eV and a threshold of 10^–7^eV for the electronic self-consistent iterations. Structural optimizations were performed using an ionic relaxation force threshold of 10^–2^ eV/Å. The out-of-plane box length of the bilayer was set to 50 Å, to avoid interactions between adjacent images. We sampled the in-plane Brillouin zone with a Γ-centered grid with a *k*-point density of 0.25 Å^–1^ using the tetrahedron method with Blöchl corrections (ISMEAR = −5). Unless otherwise noted, all binding and sliding energies were normalized by the surface area of the flat bottom layer to allow for appropriate comparisons.

Interlayer sliding calculations were performed starting from initial interlayer distances (see inset of Figure 1b for definition) corresponding to the equilibrium distances of each pristine bilyaer, as obtained by rigid vertical shifts of the initial configuration (see Figure 1b and system coordinates provided in the Zenodo database^22^). At these distances, the calculated binding energies of the GF/graphene and GF/*h*-BN bilayers were found to be approximately half of those obtained for the AB stacked graphene/graphene and C-stacked graphene/*h*-BN (where one carbon atom resides above a boron atom and another resides over the center of an *h*-BN hexagon) bilayers, respectively (see Table 1). Vertically flexible sliding calculations were performed, where following each displacement step, the vertical (*z*) coordinates of all atoms were allowed to relax.

Figure 2 presents the DFT sliding energy profiles of strained GF/graphene (filled green circles, left panels), along the *x* (armchair, upper panels) and *y* (zigzag, middle panels) sliding directions, and 45° between them (bottom panels). Notably, the sliding energy corrugation of the GF/graphene (filled green circles, left panels) contact is found to be 50–100 times (depending on the sliding direction) lower than that of aligned bilayer graphene (empty red circles). This demonstrates the effectiveness of the multicontact configuration in reducing friction of commensurate interfaces. Conversely, when comparing the sliding energy corrugation of the strained GF/*h*-BN (filled blue circles, right panels) to that of unstrained incommensurate graphene/*h*-BN (empty orange circles, see figure caption), the former is found to be at least 200 times larger. We attribute this to the disruption of the cancellation of lateral forces, characterizing the extended incommensurate contact, due to the small dimension (compared to the moiré supercell dimensions) of each individual contact in the multicontact interface. Nonetheless, for both GF contacts the sliding energy corrugation is extremely small in all sliding directions considered (<0.04 meV/Å^2^). Furthermore, unlike the flat interfaces, where homogeneous and heterogeneous contacts present significantly different friction in the aligned configuration, the multicontact GF interfaces present similar sliding energy corrugation regardless of the identity of the underlying flat surface.

**Figure 2:**
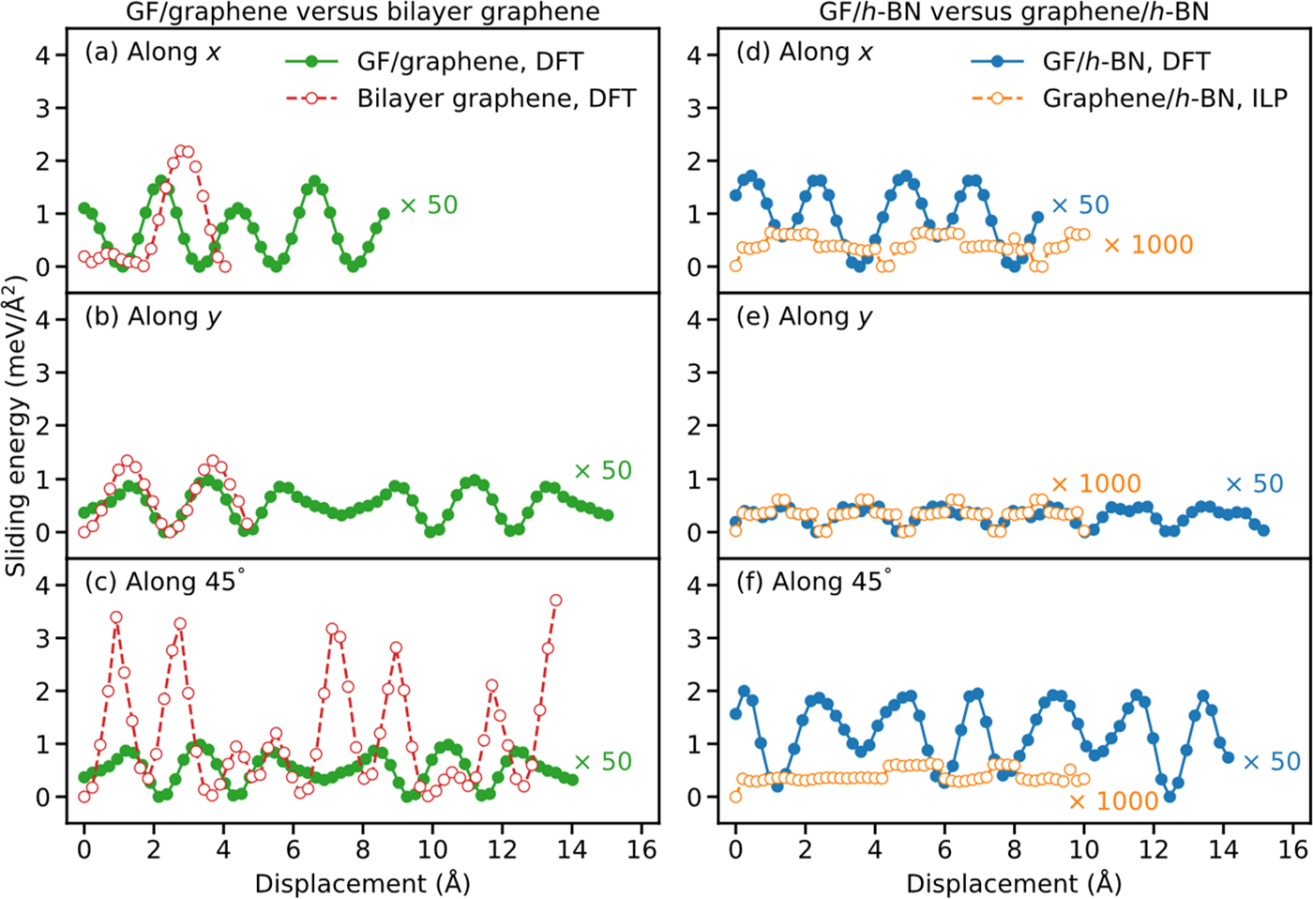
Sliding energy profiles of vertically flexible (a–c) GF/graphene (filled green circles) and bilayer graphene (empty red circles) and (d–f) GF/*h*-BN (filled blue circles) and graphene/*h*-BN (empty orange circles), calculated along the *x* (armchair, top), *y* (zigzag, middle), and in-plane 45° (bottom) sliding directions. For the graphene/*h*-BN interface, 52 × 52 graphene supercells and 51 × 51 *h*-BN supercells were used to ensure that the lattice strain in both layers does not exceed 0.1%. Due to the large supercell size, DFT calculations were not feasible in this case; hence, we performed the calculations using a dedicated classical interlayer potential (ILP) that reproduces well the DFT results.^23^ For the sake of clarity, the DFT results of the GF/graphene and GF/*h*-BN systems are multiplied by 50 and the ILP results for the graphene/*h*-BN interface are multiplied by 1000. For graphene/*h*-BN, a sliding length of 10 Å was used for all three directions. For all other bilayers, along the *x* and *y* directions, a sliding length equal to the lattice constant was used to maintain periodicity, whereas for sliding along the in-plane 45° direction, a sliding length of 14 Å was selected. All results are presented relative to the corresponding minimum energy point along each sliding profile.

As shown above, the multicontact configuration has a prominent effect on the sliding behavior of GF on flat layered substrates. Nonetheless, beyond this geometric effect chemical contributions may also have notable influence on friction. To demonstrate this, we calculated the separate sliding energy curves of the individual C_60_ molecules, comprising the GF minimal unit-cell and compared the results to the corresponding GF sliding energy profile (see Figure 3). To that end, for every snapshot along the vertically flexible GF sliding path, we cut out each C_60_ molecule and performed a single-point DFT total energy calculation. Clearly, the sum of the sliding potential curves of the individual C_60_ molecules exhibits energy barriers that are considerably higher than those presented by GF. This indicates that charge redistribution within the extended GF layer, facilitated by inter-C_60_ bonding, complements the effect of the multicontact configuration on friction.

**Figure 3:**
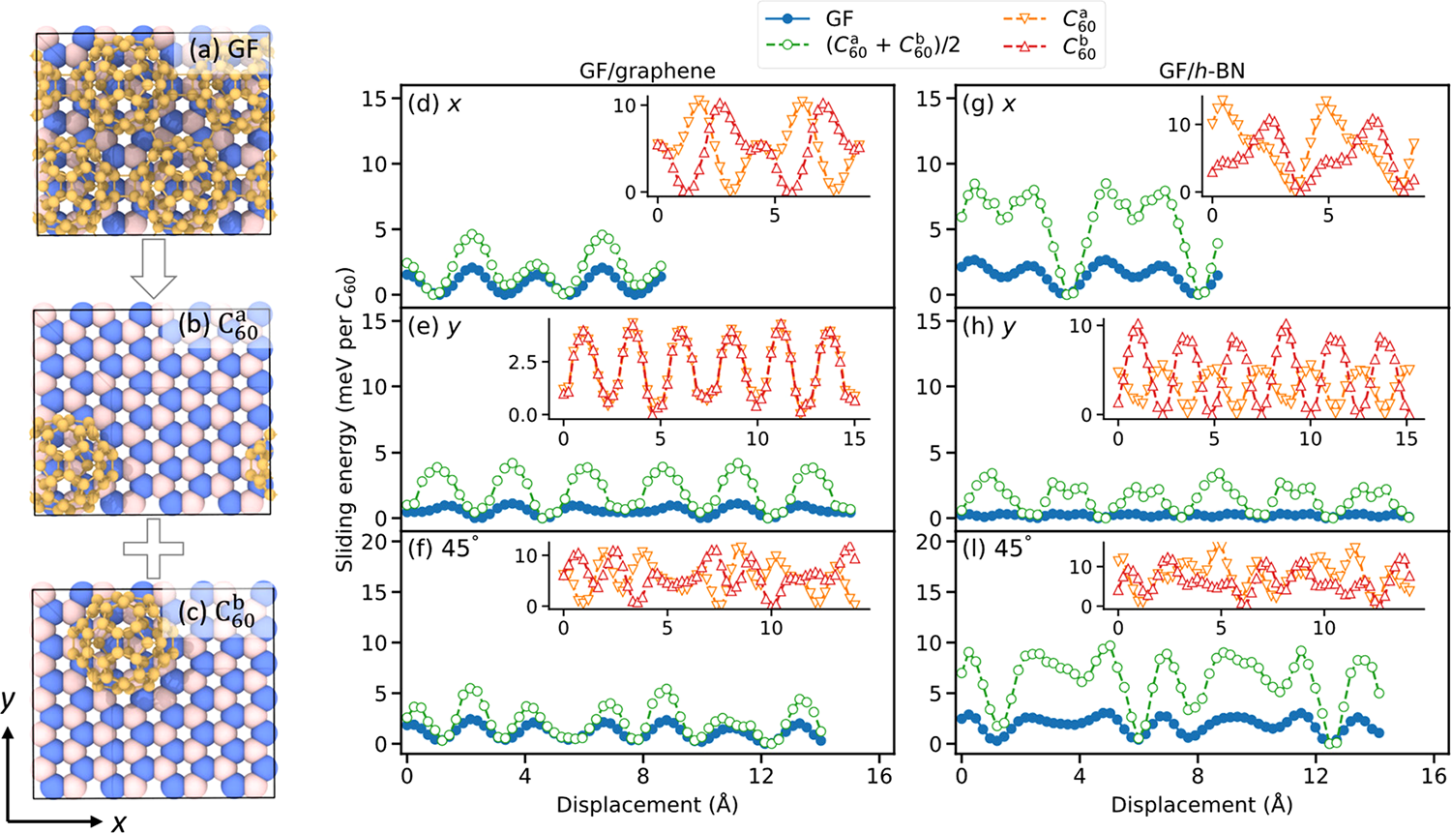
Evidence for chemical effects on friction. (a–c) Schematic representation of the individual C_60_ molecules (C_60_^*a*^ (panel b) and C_60_^*b*^ (panel c)) cut out of the GF layer (a). (d–f) Sliding energy curves (normalized per buckyball) of the two individual C_60_ molecules (empty orange and red triangles in the insets) and their sum (empty green circles) sliding on graphene, compared to the GF sliding profile (filled blue circles). (g–l) Same as panels d–f but for an *h*-BN substrate. Sliding paths along different substrate lattice directions are considered, including (d and g) the armchair (*x*) direction, (e and h) the zigzag (*y*) direction, and (f and l) 45° between them. All results are presented relative to the corresponding minimum energy point along each sliding profile.

The computational burden involved in the DFT calculations of the heterostructures considered above, dictates the consideration of relatively small supercells that exhibit an intrinsic strain of ∼3% (see Table 1). This increases the interfacial commensurability and hence enhances the corresponding sliding energy barriers and friction.^7^ To evaluate the sliding energy landscape corrugation of extended multicontact GF interfaces in the absence of in-plane strain, we resort to the registry index (RI) method,^3^ which can provide a good estimation of sliding energy profiles based on geometric consideration, in a computationally efficient manner.^24−33^ To that end, we define a circle in the *x*-*y* plane, centered around each contacting atom in the GF layer (see Figure 4a, b) and all atoms in the flat substrate layer, and calculate the sum of projected pairwise interlayer circle overlaps. Given that the sliding energy landscape is dominated by Pauli repulsions between electron clouds localized around contacting atoms in both layers, the RI of GF/graphene, is defined to be proportional to the total overlap, *S*_*GC*_, as follows:
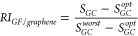
1

**Figure 4:**
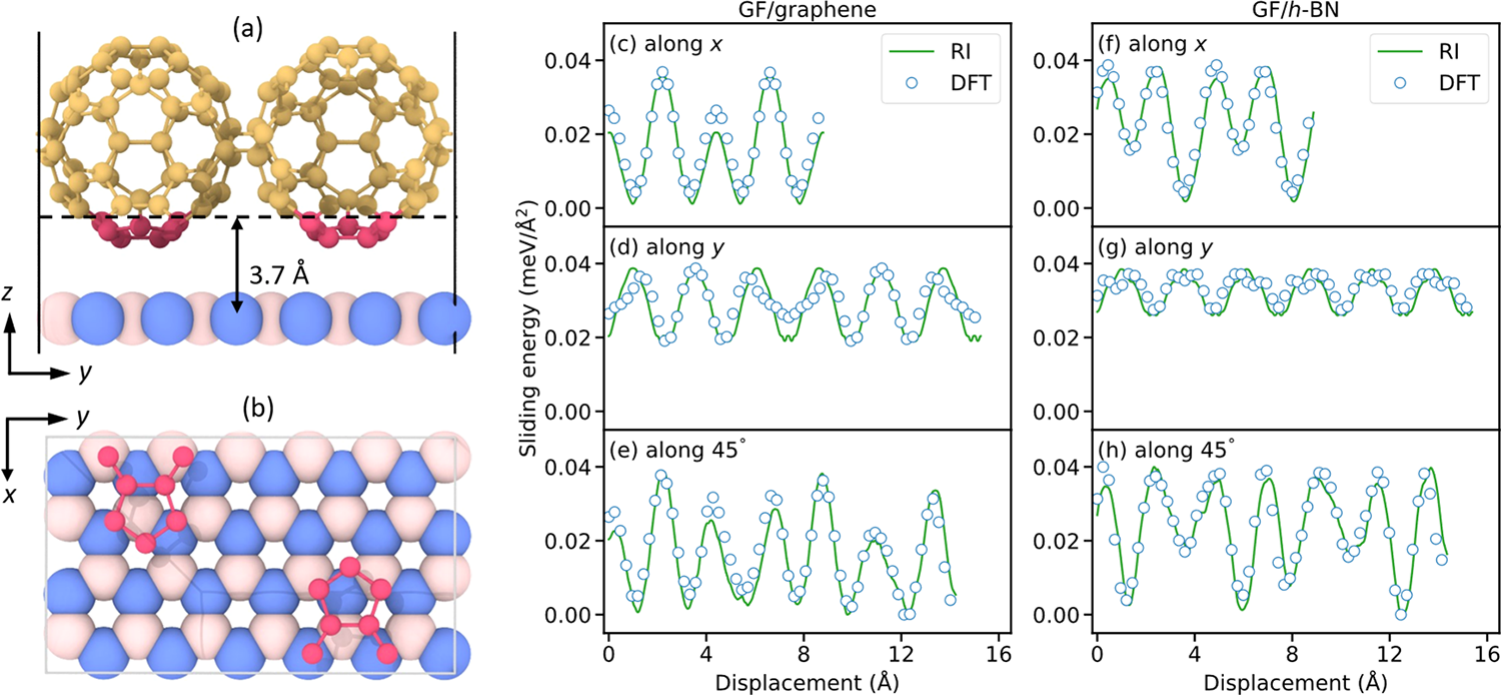
RI calculations. (a) Front and (b) top schematic views of the GF surface contacting atoms (colored red). For the RI calculations, all GF atoms residing within a vertical distance of 3.7 Å from the underlying graphene or *h*-BN surface are considered to be in direct contact with the substrate.^24,25^ Vertically flexible sliding energy profiles of (c–e) GF/graphene and (f–h) GF/*h*-BN calculated using DFT (empty blue circles) are compared to the scaled RI results (solid green lines). The DFT results are vertically shifted, such that the total energy of the lowest interlayer energy configuration of all three sliding directions considered is set as the origin. Sliding paths along different substrate lattice directions are considered, including (c and f) the armchair (*x*) direction, (d and g) the zigzag (*y*) direction, and (e and h) 45° between them. Scaling of the RI results is performed by multiplying the RI profile by a constant factor (0.0387 meV/Å^2^ for GF/graphene and 0.0400 meV/Å^2^ for GF/*h*-BN) to obtain good agreement with the DFT results.

Using the total overlap values obtained at the optimal (*S*_*GC*_^opt^) and worst (*S*_*GC*_^worst^) staking modes (in terms of interlayer energy) the RI expression is normalized to the range [0, 1],^3^ where 0 and 1 correspond to the optimal and worst stacking modes, respectively. A similar expression can be written for the GF/*h*-BN interface:
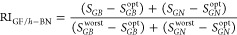
2where *S*_*GB*_ and *S*_*GN*_ are the sums of projected interlayer circle overlaps between contacting carbon–boron and carbon–nitrogen pairs, respectively.

The various circle radii are then tuned to fit the sliding RI curves of the strained systems to the shape of the corresponding DFT energy profiles (see Figure 4c-h), yielding *r*_*G*_ = 0.80 Å for the contacting GF carbon atoms, *r*_*C*_ = 0.72 Å for the graphene carbon atoms, and *r*_*B*_ = 0.23 Å and *r*_*N*_ = 0.72 Å for the *h*-BN boron and nitrogen atoms, respectively. The last three values are consistent with the radii previously reported for graphene and *h*-BN interfaces.^3^ Remarkably, the geometric RI profiles are in excellent agreement with the reference DFT predictions using a single scaling factor. Once parametrized for small (and ILP/DFT manageable) systems, the RI measure can be used to evaluate the sliding energy profiles of the extended unstrained GF/graphene and GF/*h*-BN interfaces.

To that end, we constructed GF (7 × 7)/graphene (15 × 45) and GF (10 × 13)/*h*-BN (21 × 82) heterojunction models exhibiting interlayer lattice mismatch smaller than 0.1%. Here, the numbers in parentheses represent the number of DFT-relaxed rectangular unit-cells of each layer (see Table 1) used in the *x* and *y* directions to construct the corresponding supercell. The rigid sliding scaled-RI curves (see caption of Figure 4 for the scaling factors) along the three considered sliding directions are compared in Figure 5 to the vertically flexible graphene (52 × 52)/*h*-BN (51 × 51) ILP sliding profiles. The corrugation obtained for the unstrained multicontact interfaces (0.02 μeV/Å^2^ for GF/graphene and 0.12 μeV/Å^2^ for GF/*h*-BN, which serve as upper bound for the corresponding vertically flexible results) are found to be 5–500 times smaller than those obtained for the unstrained heterogeneous graphene/*h*-BN contact. Notably, the latter itself exhibits sliding energy corrugation ∼3376 smaller than that of aligned bilayer graphene. This clearly demonstrates that by regaining lateral force cancellation, *extended* multicontact interfaces can be used to design ultrasuperlubric interfaces, where superlubricity may sustain under extreme conditions.

**Figure 5:**
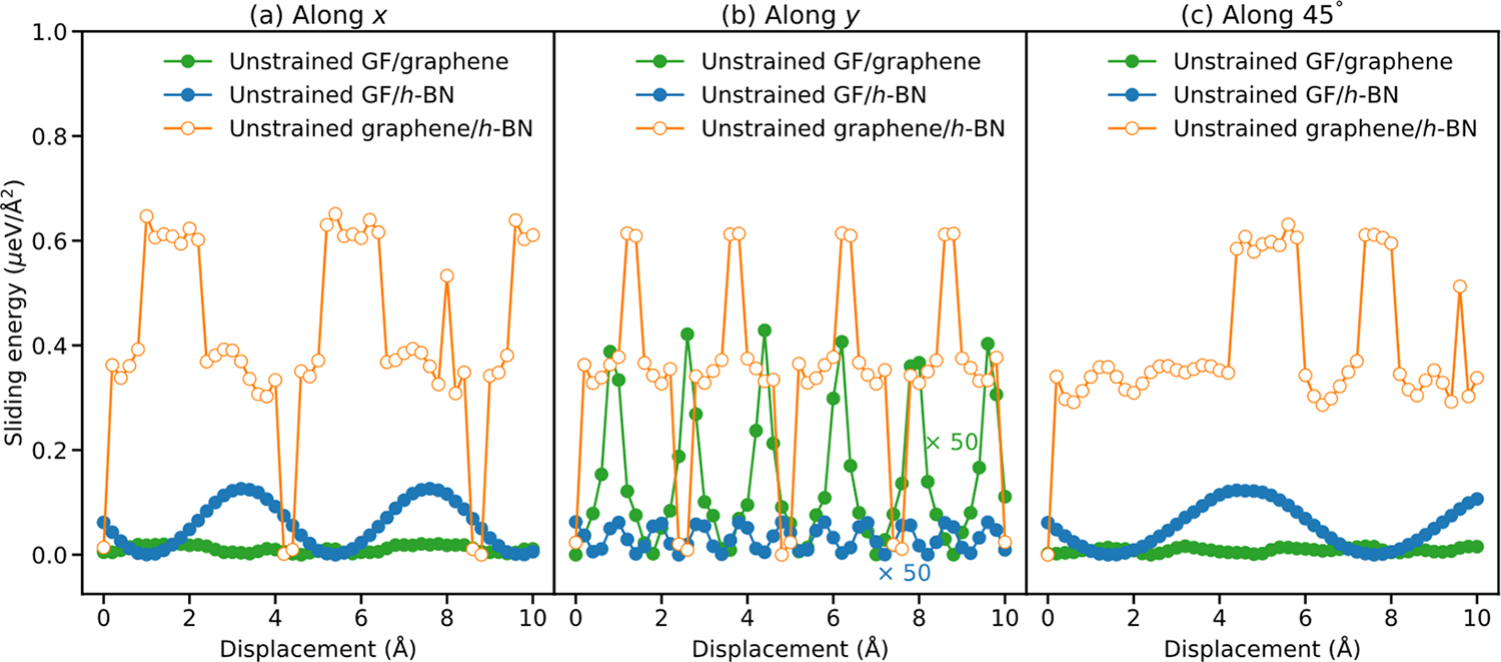
Scaled rigid sliding RI profiles of unstrained GF (7 × 7)/graphene (15 × 45) (filled green circles) and GF (10 × 13)/*h*-BN (21 × 82) (filled blue circles) interfaces, compared to unstrained graphene (52 × 52)/*h*-BN (51 × 51) vertically flexible ILP sliding energy profiles (empty orange circles). Sliding paths along different substrate lattice directions are considered, including (a) the armchair (*x*) direction, (b) the zigzag (*y*) direction, and (c) 45° between them. All results are presented relative to the corresponding minimum energy point along each profile. For the sake of clarity, the results for unstrained GF/*h*-BN and GF/graphene along the *y*-axis are multiplied by 50.

## Data Availability

Atomic coordinates and total energy traces of the interlayer sliding trajectories of the strained GF/graphene and GF/*h*-BN interfaces along the three directions considered are freely available at the Zenodo database.^22^ Any additional supporting data for this study are available from the corresponding author upon reasonable request.
